# Depression- and anxiety-related sick leave and the risk of permanent disability and mortality in the working population in Germany: a cohort study

**DOI:** 10.1186/1471-2458-13-145

**Published:** 2013-02-17

**Authors:** Felix Wedegaertner, Sonja Arnhold-Kerri, Nicola-Alexander Sittaro, Stefan Bleich, Siegfried Geyer, William E Lee

**Affiliations:** 1 Department of Psychiatry, Social Psychiatry and Psychotherapy, Hannover Medical School, Centre for Mental Health, Carl-Neuberg-Str. 1, 30625, Hannover, Germany; 2 Hannover Medical School Medical Sociology, Carl-Neuberg-Str. 1, 30625, Hannover, Germany; 3 Hannover Life Re, Karl-Wiechert-Allee 50, 30625, Hannover, Germany; 4 Department of Psychological Medicine, King’s College London Institute of Psychiatry, 10 Cutcombe Rd, London, SE5 9RJ, UK

**Keywords:** Anxiety, Depression, Treatment, Invalidity, Mortality, Occupational disability, Protection

## Abstract

**Background:**

Anxiety and depression are the most common psychiatric disorders and are the cause of a large and increasing amount of sick-leave in most developed countries. They are also implicated as an increasing mortality risk in community surveys. In this study we addressed, whether sick leave due to anxiety, depression or comorbid anxiety and depression was associated with increased risk of retirement due to permanent disability and increased mortality in a cohort of German workers.

**Methods:**

128,001 German workers with statutory health insurance were followed for a mean of 6.4 years. We examined the associations between 1) depression/anxiety-related sick leave managed on an outpatient basis and 2) anxiety/depression-related psychiatric inpatient treatment, and later permanent disability/mortality using Cox proportional hazard regression models (stratified by sex and disorder) adjusted for age, education and job code classification.

**Results:**

Outpatient-managed depression/anxiety-related sick leave was significantly associated with higher permanent disability (hazard ratio (95% confidence interval)) 1.48 (1.30, 1.69) for depression, 1.25 (1.07, 1.45) for anxiety, 1.91 (1.56, 2.35) for both). Among outpatients, comorbidly ill men (2.59 (1.97,3.41)) were more likely to retire early than women (1.42 (1.04,1.93)). Retirement rates were higher for depressive and comorbidly ill patients who needed inpatient treatment (depression 3.13 (2,51, 3,92), both 3.54 (2.80, 4.48)). Inpatient-treated depression was also associated with elevated mortality (2.50 (1.80, 3.48)). Anxiety (0.53 (0.38, 0.73)) and female outpatients with depression (0.61 (0.38, 0.97)) had reduced mortality compared to controls.

**Conclusions:**

Depression/anxiety diagnoses increase the risk of early retirement; comorbidity and severity further increase that risk, depression more strikingly than anxiety. Sickness-absence diagnoses of anxiety/depression identified a population at high risk of retiring early due to ill health, suggesting a target group for the development of interventions.

## Background

Anxiety and depression are common, overlapping mental disorders. They are important causes of absence from work and permanent disability [1-3]. Depression is one of the main contributors to disability worldwide [4]. It is a predominantly chronic recurrent disease which often leads to long periods of work incapacity [5]. Depression in working age adults generates direct costs in the field of health insurance and indirect costs from lost working hours, loss of lifetime income, and early retirement [6-8]. Depression and anxiety are particularly costly to insurers and these costs are increasing: In the past twenty years, in Germany the contribution of mental disorders to the costs of permanent disability pensions has tripled and more than half of these are caused by depression, anxiety and related neurotic disorders [9]. Accordingly this issue is of great interest to insurers and governments [10].

Common mental disorders (measured with questionnaires) have been found to be associated with increased risk of disability pension awards in representative national samples of working age adults in Denmark [11] and in a sample of a single county in Norway [12], but we know of no study examining the relationship, between people in employment taking sick leave attributed to these common mental disorders and risk of permanent disability pensioning. It is this relationship which may interest insurers because of the opportunity for future early interventions [13].

A large body of literature links depression with increased mortality [14-17]. Similar relationships have been reported whether the data were collected,by questionnaire [18,19] or by psychiatric interview [20], in the general population or in medically unwell subgroups, or in samples where ‘unnatural’ causes of death are included or excluded [20]. The issue of anxiety and mortality has less consensus: Increased anxiety has been found to be associated with increasing [21], possibly decreasing [22] mortality risks. A ‘U-shaped’ mortality curve with increasing severity [23] has also been reported. Adjustment for anxiety/depression-comorbidity is lacking in most epidemiological studies. A large study which could examine these relationships in the context of this co-morbidity, with an indication of severity such as whether the patient ever has inpatient treatment, is therefore justified.

We aimed to determine whether sick leave attributed to anxiety, depression, or both (and whether treatment was outpatient-only or incorporated one or more periods as an inpatient) was associated with elevated risk of permanent disability and of mortality in a large sample of workers in Germany who were customers of a particular health insurer.

## Methods

### Dataset

We used data from the Mettmann Regional Office of the AOK Rheinland (a German public health insurance company). The following details of insured individuals were available: data on claims for work incapacity, including diagnoses, age, sex, highest attained education, job code classification and employment status (retired or employed) and, if applicable, date of death. Workers who were insured by this company had to provide a medical certificate by the third day of absence from work. All medical reasons for work incapacity were passed on to the insurance company, which also keeps accurate data about hospital admissions and dates of death. The period of documentation extended from 1^st^ January 1987 to 31^st^ October 1996.

Insured individuals were only included in the study if they had already been insured for a period of at least 365 days and were 15 to 74 years old. Also, only subjects who had to present a medical certificate when ill were included, essentially restricting the sample to employees. Excluded cases consisted mainly of non-working spouses, older retirees and children.

Causes of death and causes of disability pensioning were unavailable to us. Therefore all-cause premature retirement and all-cause mortality were considered in relation to treatment for depression and/or anxiety, either with or without inpatient treatment.

At the time of data collection, large pension deductions made it financially unattractive to retire before age 58 without a demonstrated health reason: In this age group, disability pensions outnumber old-age pensions by 1000:1 [24,25]. At age 58 or older, the specifics of the regulatory situation in Germany at that time meant that individuals may have been declared to be retiring when a more accurate explanation would be redundancy or other non-health related causes of permanently ending work. Therefore retirement from the age of 15 to 57 years is used to describe retirement due to permanent disability in this study.

### Independent variables

Subjects were considered to have anxiety or depression if they had any of the following:

I. Depression

Major depression, single episode (ICD-9 296.2)

Major depression, recurrent episode (ICD-9 296.3)

Reactive depressive episode (ICD-9 298.0)

Neurotic depression (ICD-9 300.4)

Brief depressive reaction (ICD-9 309.0)

Prolonged depressive reaction (ICD-9 309.1)

Depressive conditions not elsewhere classified (ICD-9 311)

II.Anxiety

Anxiety states, unspecified (ICD-9 300.0)

The main independent variables of interest were 1.) anxiety/depression-related sick leave managed on an outpatient basis, in either primary care or specialist centres, and 2.) anxiety/depression related psychiatric inpatient treatment. The insured individuals were classified as “outpatient-managed sick leave” if at least one period of absence from work resulted from the above conditions without an inpatient psychiatric stay. A case of inpatient treatment was defined by at least one inpatient psychiatric stay with one of the above conditions documented (regardless of whether it was preceded or followed by sick-leave managed as an outpatient). Controls were all subjects those with neither of these events. Inpatient psychiatric treatments included treatments in specialist mixed neurological- psychiatric departments and those that began in other medical clinics and led to the patient being transferred to inpatient psychiatric treatment.

### Dependent variables

The dependent variables were: 1.) retirement before age 58, 2.) death from any cause.

### Analyses

Statistical analyses were performed with PASW/SPSS Version 18. Kaplan-Meier curves can be seen in the Additional files.

Time-at-risk started when observation began. The time-at-risk ended with the death of the participant, retirement before age 58 or when observation ended. Cox proportional hazards regression models were built to compare the mortality/disability risks of “case” participants with that of the controls (those with no [outpatient or inpatient] anxiety/depression-related sick leave at any point). We carried out separate analyses for “anxiety outpatient”, “anxiety inpatient”, “depression outpatient” and “depression inpatient” groups, each against the control group. Models were built separately for men and women and adjusted for age, education and job code classification (crude estimates are available in the Additional file 1: Table S1). Interactions between sex and the independent variables of interest were tested for. Legal basis of data transmission for the purpose of this study was section 287 of the Fifth Book of the German Social Code. No individuals were examined for this study so ethics committee approval was not sought.

## Results

### Characteristics of the sample

In total, the insurance records of 417,496 individuals were available. Of those, 4,837 had missing data, 102,102 had not been insured for a minimum of 365 days, 98,637 were under 15 or over 74 years of age, and 83,919 records pertained to individuals who were not under any obligation to present a medical certificate if they fell ill. The remaining 128,001 records were used for the analysis of mortality (Table 1). Of these 125,019 were 57 years old and younger at the beginning of the period of observation. The mean observation period was 6.4 years. The age and sex distribution as well as the proportion of employees in the sample were consistent with the distribution of employees with statutory health insurance in Germany during the period of observation [26]. Women were more likely to have outpatient-managed sick leave for depression, anxiety or both (*OR = 2.4, Χ*^*2*^ *= 644.7, p < 0.001, OR = 2.4, Χ*^*2*^ *= 631.5, p < 0.001, OR = 2.8, Χ*^*2*^ *= 313.2, p < 0.001*). The same is true for inpatient treatment (*OR = 1.4, Χ*^*2*^ *= 16.6, p < 0.001, OR = 2.3, Χ*^*2*^ *= 82.9, p < 0.001, OR = 2.0, Χ*^*2*^ *= 68.6, p < 0.001*) (Table 2). Frequencies and person years at risk for the respective subsamples can be seen in Table 2.

**Table 1:** Descriptive statistics of the sample

**Variable**	**Value**		
**Observed number**	128,001 (all employed persons)		
**Mean observation period**	2350 days (6.4 years)		
**Gender**	Male	85,502	66.8%
	Female	42,499	33.2%
**Education**	General school certificate (9 years of schooling)	40,498	31.6%
	Intermediate secondary school certificate (10 yrs of schooling)	52,208	40.8%
	Qualifying high school degree (13 years of schooling)	2,786	2.2%
	University degree	1,090	0.9%
	Not known	29,271	22.9%
**Occupation**	Occupations with little or no qualification	57,097	57.9%
	Qualified manual occupations, skilled workers	28,778	29.1%
	Qualified white-collar occupations	11,700	11.9%
	Middle-management and management positions	1,155	1.2%
	Not known	29,271	22.9%
**Age at first work incapacity with study diagnosis**	39.7 years (n = 4,611)		
**Age at first inpatient treatment with study diagnosis**	38.3 years (n = 840)		
**Age at early mortality**	54.8 years (n = 3,284)		
**Age at early retirement**	49.8 years (n = 5,282)		

**Table 2:** Sample subgroup sizes and frequency of the endpoints

	**Endpoint**		**Early retirement**			**Early mortality**		
**Gender**	**Illness**	**Type of management**	**Person years at risk**	**Number**	**Of whom retire early**	**Person years at risk**	**Number**	**Of whom die**
**Men**	**Depression**	**Outpatient**	11,672	1,551	116 (7%)	11,749	1,564	45 (3%)
		**Inpatient**	2,416	323	49 (15%)	2,426	325	24 (7%)
	**Anxiety**	**Outpatient**	11,924	1,559	75 (5%)	11,977	1,566	28 (2%)
		**Inpatient**	1,550	209	12 (6%)	1,584	213	11 (5%)
	**Both**	**Outpatient**	3,731	475	52 (11%)	3,742	477	7 (1%)
		**Inpatient**	2,166	272	39 (14%)	2,179	274	12 (4%)
	**Controls**	**None**	504,369	78,867	3,071 (4%)	521,723	81,083	2,564 (3%)
**Women**	**Depression**	**Outpatient**	13,735	1,736	120 (7%)	13,782	1,741	18 (1%)
		**Inpatient**	1,646	212	29 (14%)	1,671	216	12 (6%)
	**Anxiety**	**Outpatient**	13,159	1,725	101 (6%)	13,195	1,730	9 (1%)
		**Inpatient**	1,782	226	15 (7%)	1806	230	2 (1%)
	**Both**	**Outpatient**	5,085	624	42 (7%)	5,092	625	2 (0%)
		**Inpatient**	2,155	258	32 (12%)	2,155	258	1 (0%)
	**Controls**	**None**	234,094	36,982	1,529 (4%)	239,892	37,699	549 (1%)
**Men & women**	**Depression**	**Outpatient**	25,407	3,287	236 (7%)	25,531	3,305	63 (2%)
		**Inpatient**	4,062	535	78 (15%)	4,097	541	36 (7%)
	**Anxiety**	**Outpatient**	25,083	3,284	176 (5%)	25,172	3,296	37 (1%)
		**Inpatient**	3,332	435	27 (6%)	3,390	443	13 (3%)
	**Both**	**Outpatient**	8,816	1,099	94 (9%)	8,834	1,102	9 (1%)
		**Inpatient**	4,321	530	71 (13%)	4,334	532	13 (2%)
	**Controls**	**None**	738,463	115,849	4,600 (4%)	761,615	118,782	3,113 (3%)
		**Total**	809,484	125,019	5282 (4%)	832,973	128,001	3,284 (3%)

### Depression or anxiety as predictors of permanent disability

Risks of premature retirement are increased for all the case groups compared to controls. In multivariate adjusted analysis, persons treated for comorbid depression and anxiety in an inpatient setting had a higher risk of permanent disability (Hazard ratio (HR) 95% Confidence Interval (CI) = 4.1(3.0, 5.7) for males, HR = 3.0(2.1, 4.2) for females, see Table 3). They were followed by depression inpatients (HR[males] = 3.8(2.8, 5.0), HR[females] = 2.5(1.7, 3.6)) and outpatients in this order (HR[males] = 1.7(1.4, 2.0), HR[females] = 1.3(1.1, 1.6)). The interaction test comparing the outcome for males and females was only significant in subjects with anxiety/depression comorbidity treated as outpatients, A visual representation of this gradient can be seen in Kaplan-Meier curves (see Additional file 2: Figure S1 and Additional file 3: Figure S2).

**Table 3:** Permanent disability risks of depression and anxiety patients

**Gender**	**Illness**	**Type of management**	**Hazard ratio**	**Confidence interval**	**Significance**
**Men**	**Anxiety**	**Outpatient only**	1.178	0.937, 1.481	P = 0.161
		**Inpatient**	1.285	0.729, 2.266	P = 0.386
	**Depression**	**Outpatient only**	**1.698**	**1.410, 2.044**	**P < 0.001**
		**Inpatient**	**3.775**	**2.845, 5.009**	**P < 0.001**
	**Both**	**Outpatient only**	2.592*	**1.970, 3.410**	**P < 0.001**
		**Inpatient**	**4.134**	**3.012, 5.674**	**P < 0.001**
**Women**	**Anxiety**	**Outpatient only**	**1.299**	**1.062, 1.589**	**P = 0.011**
		**Inpatient**	1.271	0.764, 2.114	P = 0.356
	**Depression**	**Outpatient only**	**1.310**	**1.087, 1.579**	**P = 0.005**
		**Inpatient**	**2.464**	**1.705, 3.560**	**P < 0.001**
	**Both**	**Outpatient only**	1.419*	**1.044, 1.930**	**P = 0.026**
		**Inpatient**	**2.957**	**2.082, 4.201**	**P < 0.001**
**Men & women**	**Anxiety**	**Outpatient only**	**1.246**	**1.071, 1.450**	**P = 0.004**
		**Inpatient**	1.262	0.864, 1.843	P = 0.228
	**Depression**	**Outpatient only**	**1.484**	**1.301, 1.693**	**P < 0.001**
		**Inpatient**	**3.130**	**2.501, 3.917**	**P < 0.001**
	**Both**	**Outpatient only**	1.911*	**1.557, 2.346**	**P < 0.001**
		**Inpatient**	**3.540**	**2.798, 4.479**	**P < 0.001**

* Denotes significant interaction by sex at 5% level.

Bold type of results denotes statistical significance at 5% level.

Anxiety without inpatient stay was connected with a statistically significant risk of premature disability retirement HR = 1.3(1.01, 1.48). Inpatient stays for anxiety were not significantly related to increased retirement rates, but case numbers were low, which limited the power of analysis, and the estimates were similar to those of outpatients.

### Depression or anxiety as predictors for mortality

Markedly increased mortality risks compared to controls were found for patients who received inpatient treatment for depression (HR = 2.50(1.80, 3.48)). Further, of those subjects who died during the period of observation, depressed males with inpatient treatment had the lowest median age of death (47.5 years) compared to controls (57.0 years). Decreased mortality was found for female outpatients who suffered from anxiety (HR = 0.36 (0.19, 0.70)), depression (HR = 0.61 (0.38, 0.97)) or both (HR = 0.21 (0.05, 0.82)) as well as male outpatients with anxiety (HR 0.62 (0.43, 0.90)) or both depression and anxiety (HR = 0.42 (0.20, 0.88)) (see Table 4).

**Table 4:** Risk of early mortality of anxiety and depression patients

**Gender**	**Illness**	**Type of management**	**Hazard ratio**	**Confidence interval**	**Significance**
**Men**	**Anxiety**	**Outpatient only**	**0.619**	**0.427, 0.899**	**P = 0.012**
		**Inpatient**	1.579	0.873, 2.857	P = 0.131
	**Depression**	**Outpatient only**	0.910	0.677, 1.222	P = 0.529
		**Inpatient**	**2.394**	**1.601, 3.581**	**P < 0.001**
	**Both**	**Outpatient only**	**0.418**	**0.199, 0.878**	**P = 0.021**
		**Inpatient**	1.587	0.899, 2.801	P = 0.111
**Women**	**Anxiety**	**Outpatient only**	**0.362**	**0.187, 0.700**	**P = 0.003**
		**Inpatient**	0.475	0.119, 1.907	P = 0.294
	**Depression**	**Outpatient only**	**0.607**	**0.379, 0.972**	**P = 0.038**
		**Inpatient**	**2.686**	**1.514, 4.765**	**P = 0.001**
	**Both**	**Outpatient only**	**0.205**	**0.051, 0.824**	**P = 0.026**
		**Inpatient**	0.266	0.037, 1.896	P = 0.186
**Men & women**	**Anxiety**	**Outpatient only**	**0.529**	**0.383, 0.733**	**P < 0.001**
		**Inpatient**	1.182	0.685, 2.038	P = 0.549
	**Depression**	**Outpatient only**	0.798	0.621, 1.026	P = 0.078
		**Inpatient**	**2.504**	**1.802, 3.481**	**P < 0.001**
	**Both**	**Outpatient only**	**0.343**	**0.178, 0.660**	**P = 0.001**
		**Inpatient**	1.162	0.673, 2.005	P = 0.590

Bold type of results denotes statistical significance at 5% level.

The male anxiety and depression patients’ mortality advantages can most easily be seen in the middle decades of life (see Additional file 4: Figure S3). Conversely, all three curves plotting survival of patients with inpatient treatment show increased mortality risks compared to controls, for both males and females (See Additional file 5: Figure S4).

## Discussion

Three main contrasts can be seen in the results. Firstly, depression was associated with a raised risk of early retirement due to permanent disability, but anxiety had a less pronounced effect. Patients who required inpatient treatment for depression had worse outcomes than those who did not. This was not true for anxiety, where there was no compelling severity-response effect. Co-morbid depression and anxiety both had a greater effect than either disorder alone.

Secondly, male patients seemed to have a worse outcome from these disorders in terms of permanent disability than female patients, though the prevalences of both of the diagnoses and of comorbidity, was lower in males. The hazard ratio for males was significantly greater than that for females in comorbidly ill patients with outpatient-managed sick leave (though we acknowledge the problems of interpretation of interactions where both estimates are the same side of the null value [27]).

Thirdly, there were significantly *reduced* mortality risks for outpatient-only anxiety and co-morbidity in males and females, and for females only for depression.

### Depression or anxiety as predictors of permanent disability

We have shown the marked effects of depression and anxiety on participation in working life in Germany, in a large sample, where degree of impairment is indexed by whether a patient was ever treated as an inpatient or solely managed as an outpatient with sick-leave. Both outpatient sick-leave and inpatient treatments are accompanied by a elevated probability of permanent withdrawal from the workplace. For younger people suffering from depression, the consequences may be dramatic, as retirement at a young age is likely to be associated with a markedly reduced lifetime income [28].

As expected, the effect was more pronounced for patients treated on an inpatient basis (an indicator of greater severity), consistent with there being a dose–response relationship between severity of depression and both the outcomes. Also as expected, depression was diagnosed more often in women than in men (see Table 2). Retirement rates for depressed men appeared to be higher than those for women, however. And this may be because men are only put on sick leave or seek treatment for depression when they are more severely impaired [29]. ln northern European countries the way in which some men describe depressive symptoms may impair them being diagnosed with depression [30], and gender-specific misclassification to the disadvantage of male patients may also play a role [31]. Alternatively, the threshold at which help is received may be higher. Another explanation may be that personal, cultural (and medical) expectations of mood state differ between the sexes. It has been shown that health anxiety is a strong, independent, and yet under recognized risk factor for permanent disability pension [32]. We were unable to differentiate between anxiety forms, so it may be that the subsample of anxiety patients is heterogeneous. Detecting health anxiety may be a tool to differentiate at an early stage between those cases of anxiety which are more likely to lead to permanent disability.

The markedly elevated early retirement rates for both sexes after inpatient treatment suggest that inpatient services should be focussed on occupational reintegration, especially when planning the post-hospitalisation treatment phase in younger adults. Mental health workers should be aware that inpatient treatment of depression has great implications for further participation in working life, with 14% of males in our sample hospitalised with comorbid depression and anxiety permanently ending their working lives aged 57 or less.

### Depression or anxiety as predictors of mortality

The excess mortality of depression and anxiety differs very strongly dependent on study design and case definition. Hazard ratios of 1.4 for women and 1.5 for men (natural causes only – excluding suicide, homicide and accident) [18,33], and 2.1 (women) and 2.7 (men) [34] have been reported. In addition, depressed patients have highly elevated risks of unnatural deaths [35]. The estimate we found of the excess mortality in the inpatient sample here was similar, but changed when patients with anxiety were removed from the depressed sample.

It is of particular interest that depression and anxiety associated with outpatient-managed sick leave are associated with reduced mortality, seemingly in the earlier decades of life, more prominently among women, but also among men. The key to understanding this phenomenon may lie in the predominant causes of death in early decades of life. In the first five decades of life, overall mortality is low and unnatural causes of death (such as accidents, suicides and homicide) are responsible for much of the mortality, up to 30% depending on age [36]. Cardiovascular diseases and neoplasms, which are responsible for the greater part of mortality in advanced age and for which an association with depression has been demonstrated [37,38], only play a subordinate role at a young age. It is known that inpatient treatment for depression may be accompanied with sixteen fold increased mortality due to suicide [35]. This proved to be strongly linked to depression severity. There is indication that outpatient-treated depression may not be associated with increased suicide rates [39]. High trait anxiety (on several measures) has been found to be associated with reduced mortality in younger British born adults, and increased mortality later in life, following the pattern proposed above. Deaths from injuries are reduced in younger participants, and deaths from medical causes are later increased [40], and these aspects may explain these differences in mortality between strata.

It is possible that mild depression, or its treatment with antidepressants, has a causal effect on mortality. Here, presumably depressed individuals behave differently to their well counterparts, by taking fewer direct risks and possibly caring for their health more diligently. This could be driven by seeing their situation with ‘Depressive Realism’ [41]), or simply that the inactivity seen in depressed people may be incidentally protective in that age group. Finally there is the reported cardioprotective effects of the class of antidepressant drug known as specific serotonin uptake inhibitors (SSRIs) which may play a role [42].

### Strengths and weaknesses

#### Representativeness

The observations were made in a West-German population during a period that saw both a phase of economic boom and a phase of stagnation. There were no major legal changes in access to retirement pensions during the observation period, so cohort effects whereby major legal changes could affect both diagnosis of mental disorder and permanent disability/mortality are unlikely. Similarly, trends in mental disorder prevalence and permanent disability incidence could create spurious findings in a cross-sectional study, a prospective cohort study such as this is not vulnerable to such effects Between eighty-five and ninety percent of all inhabitants of Germany have statutory health insurance so the used data set had high representativeness. The dataset was also largely complete with very few (<1%) missing data-points. Not only was every health service usage episode taken up in the observation period documented, but also the most important social data such as occupation, education, level of income, type of income and, where appropriate, date of retirement and death are routinely collected and so were available. Because payment for organisations and access to care for individuals depends on collection of these data, they were likely to be diligently recorded. The main advantages of this method are the large case numbers and the fact that the complete dataset effectively includes the control group. Therefore there was no differential recruitment which may have caused recruitment bias. A particular advantage was also being able to record very short work incapacity periods here, which made it possible to detect milder episodes of illness.

An explanation for the apparent advantages of depression and anxiety on mortality (but not early retirement) in younger decades could also be a ‘healthy presenter’ effect whereby the depressed younger people who attend their doctors are physically healthier than other depressed people who do not. This is in contrast to the inpatient group, where one expects nearly all people with depression of this severity to be included. This assumption requires the established relationships of physical ill-health with depression [33,43-45], and also that between primary-care attendance and depression [46], to be absent, reversed or overwhelmed in the younger part of this sample. Alternatively patients with additional risk factors for early mortality may be more likely to be treated as inpatients than outpatients due to poor physical health, home circumstances, comorbidity with alcohol problems, and other factors.

Another source of error may be the control group. In the group of people who had never had a any time off work for depression and anxiety may be people who are avoidant of healthcare in general. These individuals may suffer increased mortality later in life because of this rather than their ‘non-diseased’ status.

#### Participation biases

As a result of the social structure of the employees insured by the AOK, the insured individuals as a whole differ from the general population with regard to social status and disease burden [47]. Occupations involving simple manual jobs and individuals with a low to moderate level of education were predominant. With regard to gender distribution, there is a slight displacement in favour of men in the sample. In the working population, the ratio of men to women in the period of data collection was 60:40 [26]. The former effect is likely to cause an underestimate of any association because of the observation that low social status is accompanied by an increased disease burden and resulting disability pension award. Further, our estimates are consistent with the literature, though they are not always significant [48,49]. Another weakness is that this study did not include people who were unemployed, as they were not included in the study sample. These people were excluded, because they were not obliged to present a medical certificate if taken ill, so no diagnostic data were available.

#### Information biases

Is unknown to what extent doctors did not issue a depressive/anxiety diagnoses during sick leave for fear of stigmatization of the patient, or whether this was differential by gender [31]. There may therefore have been cases which were not detected by our methods, but the prevalence of being depressed in this sample was 2.4% for men and 5,3% for women, suggesting not many cases were missed, taking into account the low prevalence of psychiatric disorders expected in a working population. Nonetheless many depressive symptoms are missed in primary care [5], and it is likely that the we missed some cases of mild disorder. This may have caused us to overestimate the effects because of the under-representation of the milder cases of disorder, which presumably are less related to the outcomes.

#### Limitations of secondary data analysis and statistical power

Given case numbers in the hundreds of thousands, it became clear in this study that due to restriction and low event frequency the individual categories quickly became so small that further stratification was not appropriate. Another disadvantage was that, as is inevitable in a cohort study, causation cannot be determined unequivocally from the dataset because of residual or unconsidered confounding.

An intrinsic problem of secondary data analysis of statutory health insurance data is that different doctors have different thresholds for applying the same diagnostic code [50]. Although inpatient treatments and work incapacity diagnoses have been an inherent part of health reporting for many years, diagnostic quality can only be regarded as sufficient on the level of large disease entities [51]. Thus we used large disease entities as the exposure measures for this study.

As has been noted, the control group included subclinically depressed or anxious patients, those who seek treatment but have no sick leave because of their disorder, and those who attended their doctors with depression or anxiety but were diagnosed with something else for whatever reason. Subclinical depression (causing no identified sickness absence from work) may have an effect on early retirement [52,53] but its prevalence is unknown, not only in this sample. So it is impossible to deduce the additional risks associated with depression and anxiety as a whole for mortality and permanent disability from the parameters used in this study. The point prevalence of depressive disorders in Germany was stated as 6.4% in the last systematic survey in the German-speaking countries in 1998 [54], compared with 4.2% in Europe in 2003 [55]. Newer data suggests a point prevalence of depression in Germany of 8.6% for women and 4.5% for men [56]. These figures are compatible with there having been no trend in depression prevalence in Germany in recent years. In the sample of this study the six-year prevalence was 2.4% for men and 5,3% for women. The differences are explicable by the tighter criteria in this study, but they suggesting there are many cases of depression which did not cause identified sickness absence, leading to an underestimation of the effect of anxiety and depression as a whole.

The probability of early retirement occurring is largely affected by legal regulations. For this reason it was important for the authors to choose an observation period that was before the abolition of the statutory occupational disability pension, which was the 1^st^ January 2001 and changes in the interaction of unemployment benefits and retirement for older employees on 1^st^ January 2003. Diagnostic data from outpatient treatment – and not only outpatient-managed sick leave - have been reported to the health insurance companies again in relation to individual insured persons since 2004 and the implementation of the two bodies of legislation has been completed, there is a desire for more differentiated analysis with newer data. However, the endpoints used here can only be used again with restrictions: The further the abolition of the statutory occupational disability insurance lies in the past, the less the pension claims statistics reflect real burden of permanent disability, which is now increasingly removed from the official statistics as a privately insured issue.

## Conclusions

We were able to find a strong dose–response relationship of disease management mode and anxiety/depression comorbidity with permanent disability. While depression appeared to influence mortality and permanent disability in the same direction, this was not the case for anxiety. To an unknown extent, lifestyle factors, service use and physical comorbidities influenced disablement or death in the present study. A better insight into the causal relationships between depression, anxiety and outcome, with particular focus on the positive outcomes, is necessary and warrants further study. No increased mortality could be determined for patients who only had outpatient-managed sick leave for depression or anxiety, which means that we found no reason for these patients to be treated differently by life-insurers.

Statutory health insurance data on anxiety and depression cases associated with sick leave is useful to identify people at elevated risk of disability or mortality who might not otherwise come to clinical attention. Health policy-makers, increasingly interested in preventing sickness-absence from becoming long-term or permanent may like to consider testing interventions focused on preserving and restoring occupational functioning, in groups presenting requests to be certified as unfit for work with depression and/or anxiety.

## Competing interests

The authors declare that they have no competing interests. The study was funded by the Hannover Rückversicherung AG.

## Authors’ contributions

FW conceptualized the study, wrote the manuscript, defined the statistical methods and did the calculations. SAK prepared the dataset and did the first data analysis. NAS had the first idea for the study, interpreted the results, corrected the manuscript. SB provided computer resources. SG provided the dataset for analysis and revised the manuscript. WEL co-wrote the manuscript, gave the most important ideas for additional statistical calculations and revised the manuscript. All authors read and approved the final manuscript.

## Pre-publication history

The pre-publication history for this paper can be accessed here:

http://www.biomedcentral.com/1471-2458/13/145/prepub

## Supplementary Material

Additional file 1: Table S1 Crude and partially adjusted estimates. Click here for file

Additional file 2: Figure S1 All-cause permanent disability pensioning – Kaplan-Meier curves for the male sample. Click here for file

Additional file 3: Figure S2 All-cause permanent disability pensioning – Kaplan-Meier curves for the female sample. Click here for file

Additional file 4: Figure S3 All-cause mortality – Kaplan-Meier curves for the male sample. Click here for file

Additional file 5: Figure S4 All-cause mortality – Kaplan-Meier curves for the female sample. Click here for file
