# Supplementary figures and images for: Depression- and anxiety-related sick leave and the risk of permanent disability and mortality in the working population in Germany: a cohort study
Source: BMC Public Health. 2013 Feb 17;13:145. doi: 10.1186/1471-2458-13-145 (PMC3698165; doi:10.1186/1471-2458-13-145)

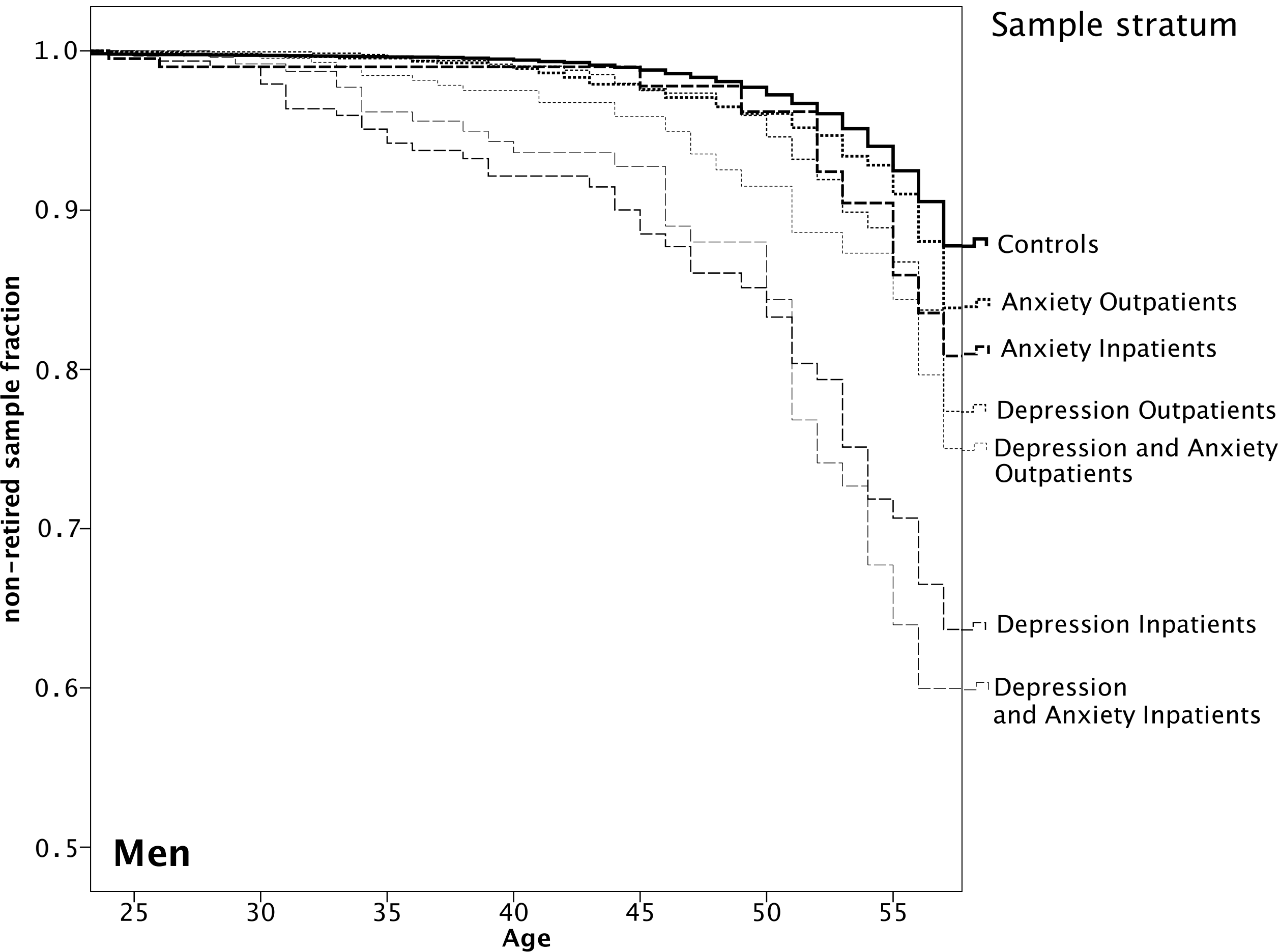

Supplement: Additional file 1: Table S1 — Crude and partially adjusted estimates. [file 1471-2458-13-145-S1.pdf]

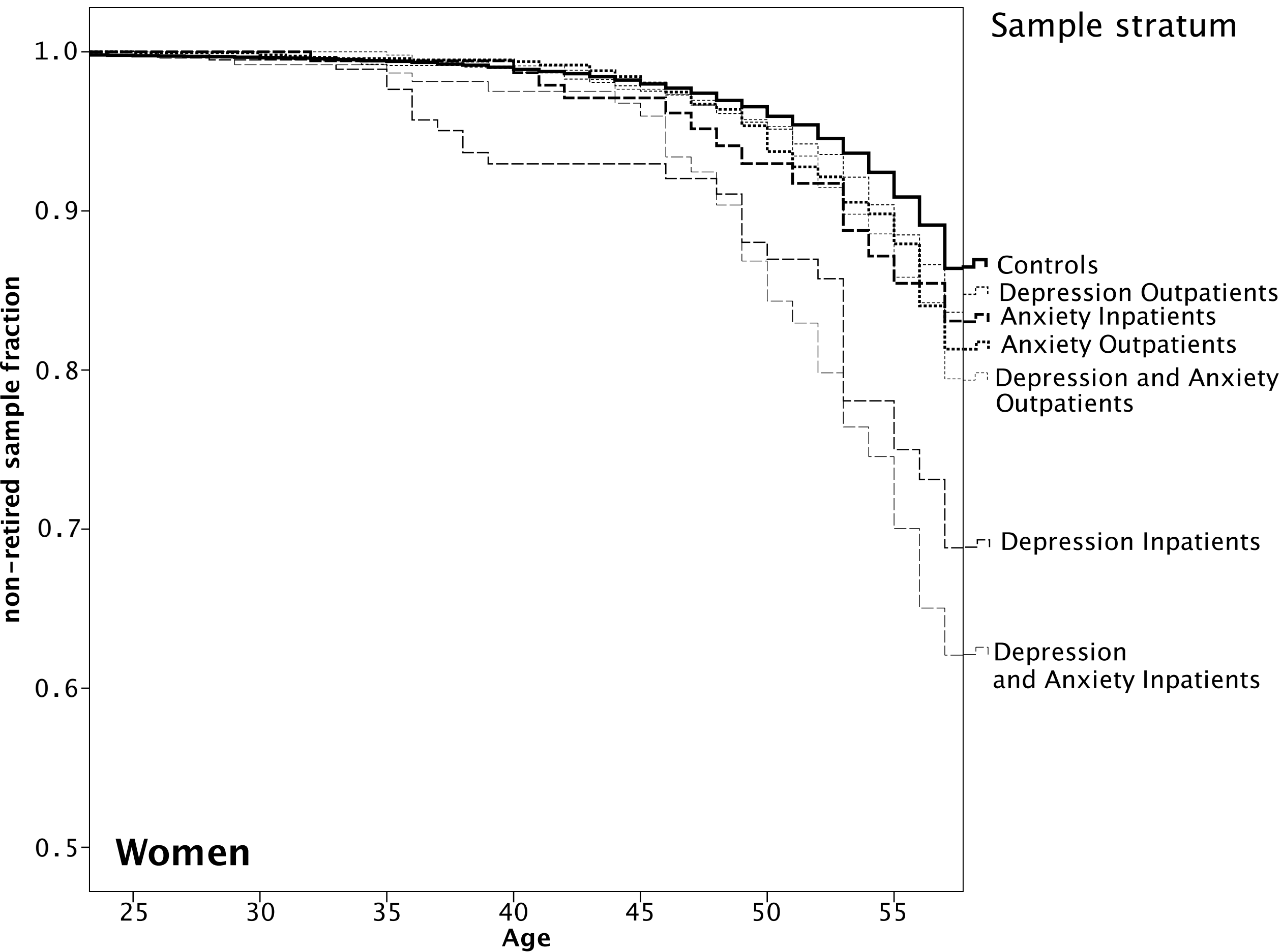

Supplement: Additional file 2: Figure S1 — All-cause permanent disability pensioning – Kaplan-Meier curves for the male sample. [file 1471-2458-13-145-S2.pdf]

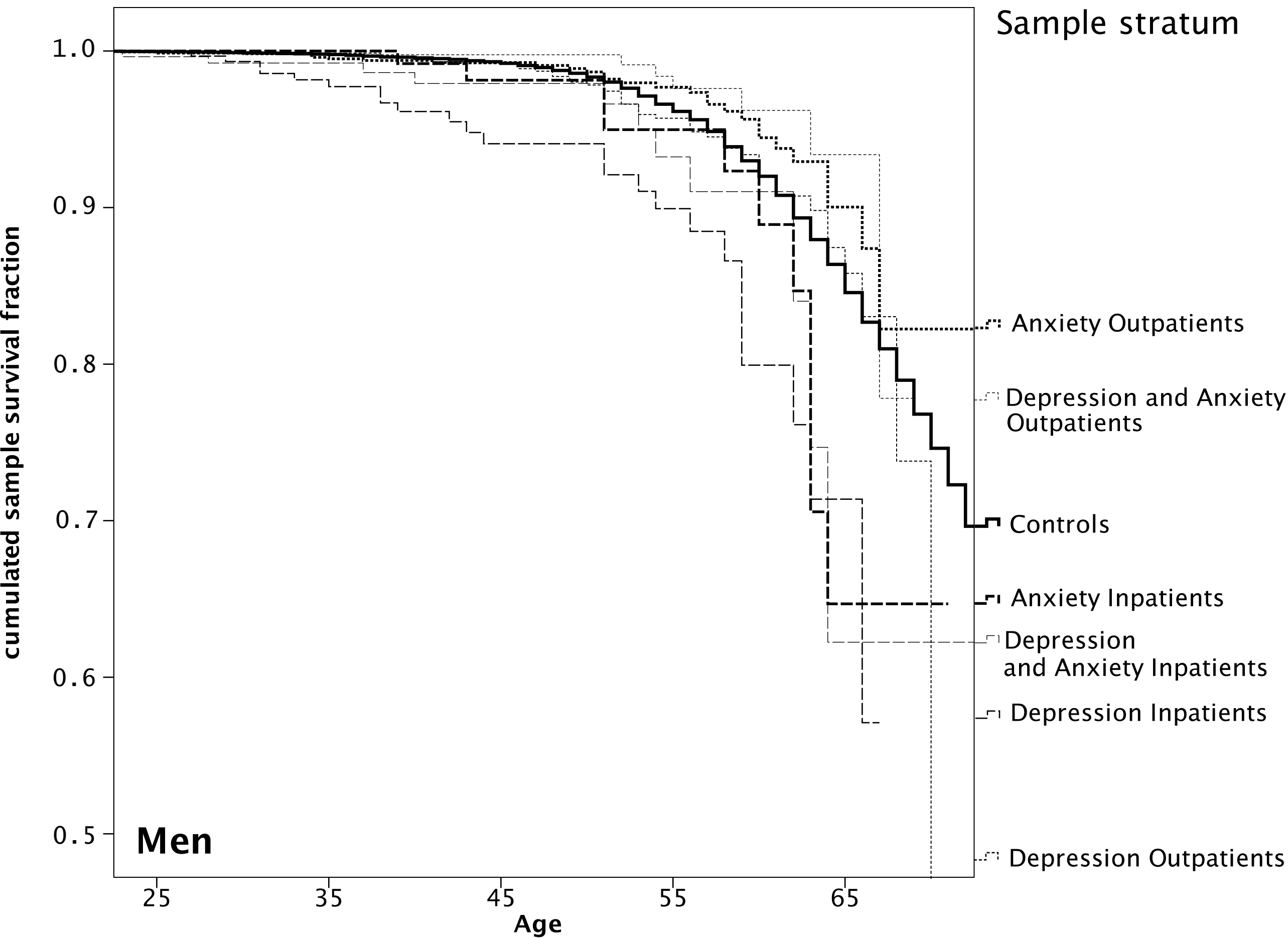

Supplement: Additional file 3: Figure S2 — All-cause permanent disability pensioning – Kaplan-Meier curves for the female sample. [file 1471-2458-13-145-S3.pdf]

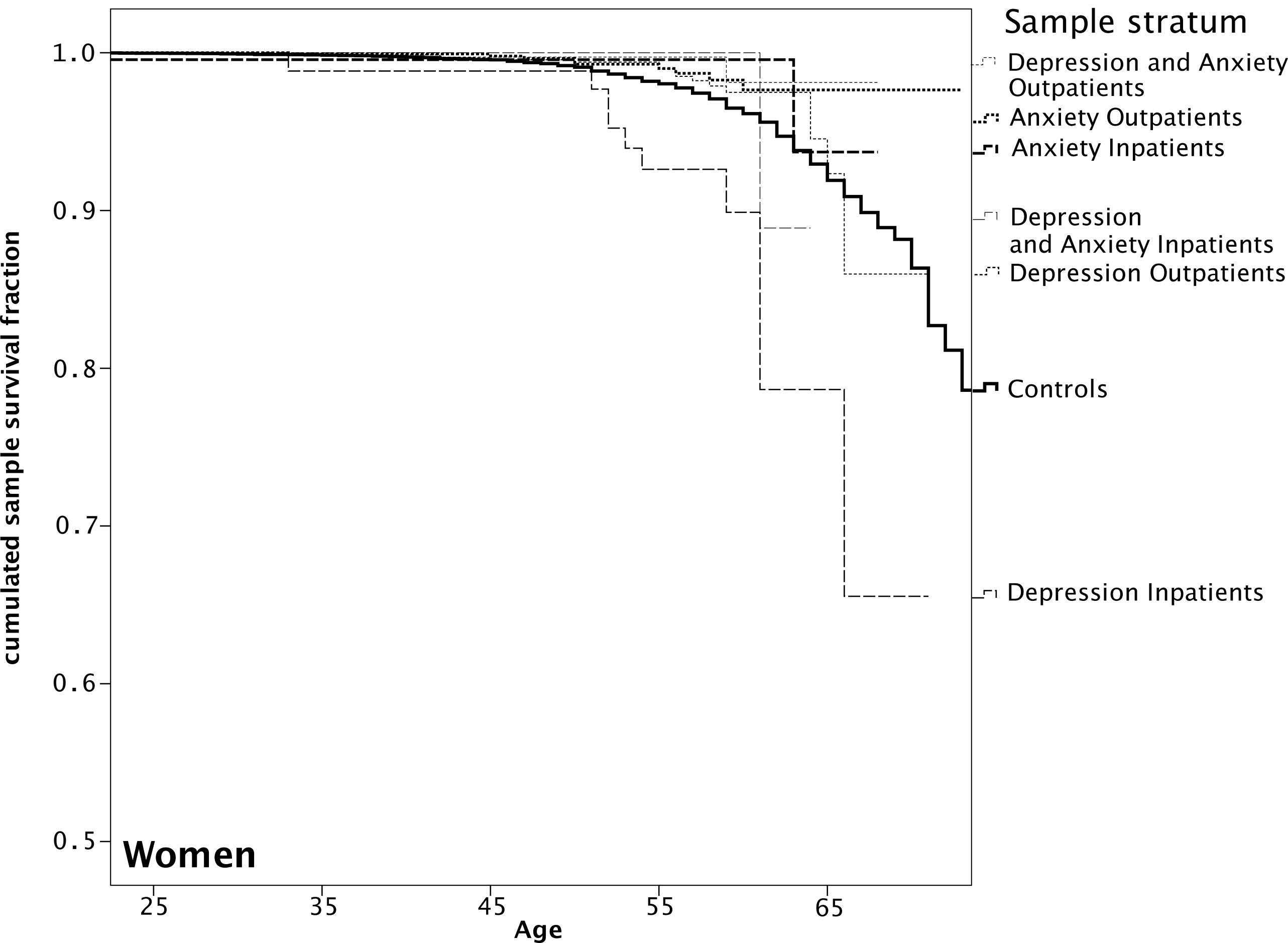

Supplement: Additional file 4: Figure S3 — All-cause mortality – Kaplan-Meier curves for the male sample. [file 1471-2458-13-145-S4.pdf]
